# 715 The incidence of refeeding syndrome in burn patients receiving enteral nutrition

**DOI:** 10.1093/jbcr/irac012.269

**Published:** 2022-03-23

**Authors:** Bryce K Hendren-Santiago, Sebastian Q Vrouwe, Mikhail Pakvasa, Valerie Reynolds, Dejan Micic, Lawrence J Gottlieb

**Affiliations:** The University of Chicago Pritzker School of Medicine, Chicago, Illinois; University of Chicago, Chicago, Illinois; UC Irvine, Coromandel Mar, California; University of Chicago Medicine, Evanston, Illinois; University of Chicago Medicine, Chicago, Illinois; University of Chicago, Chicago, Illinois

## Abstract

**Introduction:**

A major burn injury result in a hypermetabolic response which leads to increased nutritional requirements. Previous studies have shown that burn patients experience severe electrolyte abnormalities that are likely the result of multiple factors, but share similarities to refeeding syndrome (RS). Recently, the American Society for Parenteral and Enteral Nutrition (ASPEN) published consensus recommendations on RS, including clinical definitions and a grading system. Our aim was to evaluate this definition in a cohort of burn patients.

**Methods:**

A retrospective analysis was performed of adults admitted to an American Burn Association-verified center from 2015-2020 with a burn injury who received enteral feeding for at least 72 hours. Patients with a length of stay < 7 days were excluded. Patients were categorized as having mild, moderate, or severe RS based on the newly established ASPEN criteria, defined by a decrease in any one, two, or three of serum phosphorus, potassium, and/or magnesium levels from baseline by 10%–20% (mild RS), 20%–30% (moderate RS), or >30% and/or organ dysfunction resulting from a decrease in any of these and/or due to thiamin deficiency (severe RS).

**Results:**

A total of 350 patients were reviewed, from which 72 met the inclusion criteria. The mean patient age was 52 ± 18 years, and the average BMI was 28.3 ± 7.4. The average burn size was 21.7 ±17.0% TBSA and the average length of stay was 36 ± 32 days. A total of 57 (89%) patients had severe RS while five (8%) exhibited moderate and two (3%) developed mild refeeding syndrome. Of the 57 patients with severe RS, hypophosphatemia was the most prevalent (89%) followed by hypokalemia (19%) and hypomagnesemia (13%). Regression analysis comparing those with severe RS to those with mild or moderate RS revealed that number of surgeries performed was positively associated with the development of severe RS (p=0.0006). %TBSA trended but was not statistically significant (p=0.063). All other variables evaluated were not significant on univariate analysis.

**Conclusions:**

The presence of severe electrolyte abnormalities as a result of burn injuries is significantly more common than originally anticipated. Further investigations are needed to determine if these abnormalities truly reflect RS or are primarily a result of the hypermetabolic response characteristic of burn patients.

